# Participation in Community Group Activities Among Older Adults: Is Diversity of Group Membership Associated With Better Self-rated Health?

**DOI:** 10.2188/jea.JE20170152

**Published:** 2018-11-05

**Authors:** Masayoshi Zaitsu, Ichiro Kawachi, Toyo Ashida, Katsunori Kondo, Naoki Kondo

**Affiliations:** 1 Department of Public Health, Graduate School of Medicine, The University of Tokyo, Tokyo, Japan; 2 Department of Social and Behavioral Sciences, Harvard T.H. Chan School of Public Health, Boston, MA, USA; 3 Graduate School of Economics, The University of Tokyo, Tokyo, Japan; 4 Department of Social Preventive Medical Sciences, Center for Preventive Medical Sciences, Chiba University, Chiba, Japan; 5 Department of Gerontological Evaluation, Center for Gerontology and Social Science, National Center for Geriatrics and Gerontology, Obu, Aichi, Japan; 6 Department of Health and Social Behavior/Department of Health Education and Health Sociology, School of Public Health, The University of Tokyo, Tokyo, Japan

**Keywords:** social capital, gender, social activities, sport

## Abstract

**Background:**

Participation in community activities (eg, sports and hobby groups or volunteer organizations) is believed to be associated with better health status in the older population. We sought to (1) determine whether a greater diversity of group membership is associated with better self-rated health and (2) identify the key dimension of the membership diversity (eg, gender, residential area, or age).

**Methods:**

We performed a cross-sectional study of 129,740 participants aged 65 years and older who were enrolled in the Japan Gerontological Evaluation Study in 2013. We assessed the diversity of group membership using (1) a continuous variable (range 0–4) accounting for the total degree of each diversity dimension or (2) dummy variables for each dimension. We estimated prevalence ratios (PRs) and 95% confidence intervals (CIs) for better self-rated health according to the diversity of group membership, using Poisson regression and robust variance with multiple imputation, adjusted for other covariates.

**Results:**

The participants involved in social groups with greater diversity had better self-rated health: the PR per one point unit increase in diversity was 1.03 (95% CI, 1.02–1.04). Participation in gender-diverse groups was associated with the best profile of health (PR 1.07; 95% CI, 1.04–1.09).

**Conclusions:**

Among the older population in Japan, higher group diversity is associated with better self-rated health. Gender is the key dimension of diversity that is associated with better self-rated health.

## INTRODUCTION

Among aging individuals, social participation has been shown to promote better maintenance of activities of daily living and cognitive function, as well as self-rated health.^1^^–^^6^ Social participation provides individuals with access to various resources, such as heath-relevant information and emotional and instrumental support. Previous studies have shown that participation in “horizontal” organizations consisting of peers, such as sports and hobby groups and voluntary associations, may be particularly effective in the prevention of the onset of functional limitations and disability among older adults.^2^^–^^4^

However, few studies have focused on whether the membership composition (or “diversity”) of these groups/organizations has an influence on health. In research on social capital, which can be defined as “resources that are accessed by individuals as a result of their membership of a network or a group,” participation in community activities/groups is a key activity of community residents to increase their social capital, and the types of such groups are often characterized as “bonding” and “bridging” types.^7^ Groups that are homogeneous of membership characteristics (eg, men’s only clubs, or groups that only include members from one social stratum) may provide strong bonding relationships based on shared identity, but they may not be as effective in mobilizing resources for its members (bonding social capital). For example, a homogeneous group with bonding social capital was not protectively associated with dental health status in an older population in Japan.^1^ Groups with membership that “bridges” diverse social characteristics (ie, heterogeneity in membership characteristics) are therefore more efficient in enabling individuals to access resources (bridging social capital).

To our knowledge, only one previous study has looked at whether the diversity of group membership is associated with better health outcomes. In a study based in a city in western Japan, Iwase et al^8^ asked residents (aged 20–80 years) to rate the diversity of six different types of associations that people participated in: Parents and Teachers Associations, sports clubs, alumni associations, political campaign clubs, citizen’s groups, and community organizations. High bridging social capital was associated with better health compared with those who reported no participation (OR 0.25; 95% CI, 0.11–0.55).^8^ However, the association between the levels of group diversity and health is unclear. In addition, no study has investigated which key dimensions of diversity (eg, age, gender, and residential area) are most strongly associated with health.

Therefore, in the present study, we sought to determine (1) whether a higher level of group diversity is associated with members’ better self-rated health and (2) which dimension of the diversity is most strongly associated with self-rated health in community-dwelling seniors.

## METHODS

### Data source

We used a cross-sectional dataset of the Japan Gerontological Evaluation Study (JAGES) in 2013. The JAGES is an ongoing, population-based cohort study of healthy people aged 65 years and older across Japan,^9^ and details of the study (JAGES2013v3) have been described elsewhere.^10^^–^^12^ Briefly, the cohort sampled community-dwelling seniors residing in 30 municipalities in 14 prefectures across Japan. We mailed self-reporting questionnaires to the older population (aged ≥65 years) in those municipalities.^10^^,^^11^ Using public residential registers, we conducted a census of residents aged 65 years and older in areas where the population was less than 5,000 (13 municipalities) and a simple random sample of municipalities where the population was 5,000 or more (16 municipalities). We mixed the sampling methods in one municipality. Responses were obtained from around 138,000 individuals in 2013 (response rate, 70.3%).^10^ The Ethics Committee on Research of Human Subjects at Nihon Fukushi University approved the JAGES protocol (Protocol Number: 13-14). Informed consent was presumed when respondents returned the mailed questionnaires.

### Outcome

Our main outcome was self-rated health (poor, fair, good, or excellent) as assessed by the question, “how is your current health status?” We chose self-rated health for the outcome because self-rated health is a strong predictor of mortality and hospitalization.^13^ We binarized the self-rated health (poor/fair versus good/excellent).

### Diversity of group membership

The survey asked respondents whether they participated in different horizontal organizations, such as sports group, volunteer group, and hobby group. A horizontal group was defined as a group with an organizational structure that does not involve hierarchy (ie, it is based on relationships among equals).^1^ We specifically focused on the association between health and the diversity of group membership in sports groups, volunteer groups, and hobby groups because previous studies suggested that participation in these groups was particularly protective for health status, but participation in other groups (eg, senior citizen clubs, neighborhood associations or residents’ associations, study or cultural groups, nursing care prevention or health-building activities, teaching skills or passing on experiences to others, local events, looking after older people, assistance for seniors, child-rearing support, and local environmental improvement) was not associated with health.^1^^–^^4^ In a priori analysis, we confirmed that these groups did not show significant associations with health (data not shown).

We asked respondents who said that they participated in these organizations to rate further the diversity of the membership of the group in which they participated most frequently. The dimensions of diversity in basic demographic characteristics were gender ratio, area of residence, and age composition, defined as follows.

1. Gender ratio: women or men only (not diverse); mixed (diverse).
2. Area of residence: Only people from the same municipality (not diverse); some people from other municipalities (diverse).
3. Age composition: mostly people of the same generation (not diverse); mixtures of different generations, with a difference in age of at least 20 years (diverse).

Using this information, we categorized the respondents into five mutually exclusive groups and created a continuous variable (ranging 0–4): level 0 (not participating in any group), level 1 (involved in groups, but the group is not diverse in any of the three dimensions), level 2 (involved in groups, and one of the three dimensions was diverse), level 3 (involved in groups, and two of the three dimensions were diverse), and level 4 (involved in groups, and all three dimensions were diverse). We also created dummy variables for each dimension of the diversity (gender, residential area, and age).

### Covariates

We included the following covariates in our models based on previous studies^1^^,^^4^^,^^14^^,^^15^: age, gender, current working status (yes/no), marital status (single, married, divorced/widowed, or others), cancer comorbidity status (yes/no), cardiac disease (yes/no), stroke (yes/no), and residential area (535 small areas of school/community comprehensive care center districts), equivalent annual household income (<1.5 million yen, 1.5–2.4 million yen, or ≥2.5 million yen), educational attainment (<6 years, 6–9 years, 10–12 years, or ≥13 years).

### Statistical analysis

We performed multiple imputation for missing data among 129,740 participants using all data, including self-rated health, the diversity level, and all the other covariates. We generated five imputed datasets with Multiple Imputation by Chained Equations (MICE) method.^16^ We used the user-written command “ice” written by Patrick Royston (available from http://www.stata-journal.com/software/sj9-3/) in STATA.

Next, we estimated the prevalence ratio (PR) and 95% confidence interval (CI) of better self-rated health according to the diversity level (the continuous variable), using Poisson regression with robust variance (model 1).^17^ We chose the continuous variable to create the composite measure that represents the overall levels of group diversity. A priori analysis with the categorical variable of the diversity levels showed a similar trend (data not shown). We combined the five PRs and 95% CIs obtained at each imputed dataset into one combined PR and 95% CI. We adjusted for age, gender, current working status, marital status, comorbidity, residential area, income, and education. We also estimated the PR and 95% CI according to each dimension of diversity, adjusted for the same covariates (model 2).

For subgroup analysis, we performed stratified analysis to estimate the PR and 95% CI with those involved in each group (sports, volunteer, hobby, or other groups) compared with those not involved in any groups. We also performed a sensitivity analysis of those who provided complete data (59,907 participants).

## RESULTS

**Figure 1.**
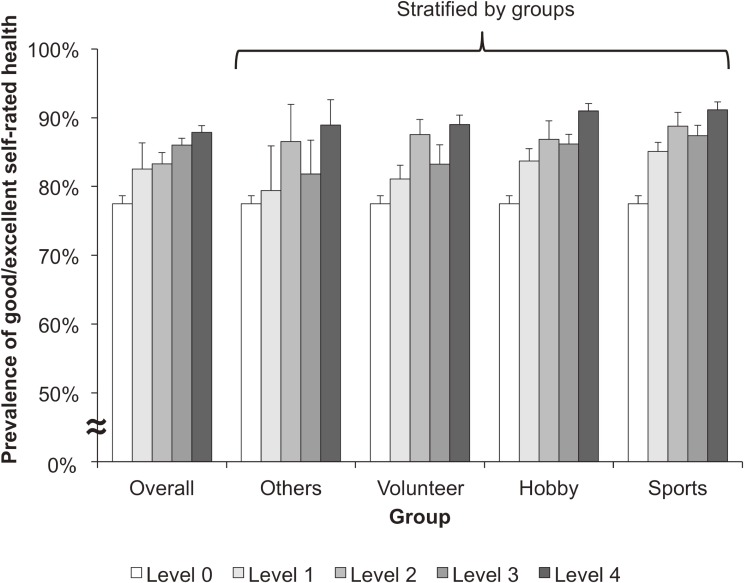
Prevalence of good/excellent self-rated health for each diversity level of group membership. Each bar chart and error bar shows a combined prevalence and 95% confidence interval of good/excellent self-rated health estimated by multiple imputation. We assessed the diversity level of group membership based on three dimensions of diversity (gender, residential area, and age composition). The diversity level was graded into as follows: level 0 (not involved in any groups), level 1 (involved in a group; none of the three dimensions was diverse), level 2 (involved in a group; one of the three dimensions was diverse), level 3 (involved in a group; two of the three dimensions were diverse), or level 4 (involved in a group; all dimensions were diverse).

A total of 129,740 participants in the JAGES in 2013 were analyzed. The following missing data were multiply imputed: self-rated health (4,505), information on groups or diversity dimensions (48,084), working status (13,297), marital status (2,917), comorbidity status (8,667), equivalent annual household income (26,093), and educational attainment (2,987). The characteristics were similar between those who completed the information on diversity and those who had any incomplete data on diversity (eTable 1). Compared with those who have poor/fair self-rated health, the overall diversity level and the prevalence of participating in gender-diverse groups were higher in those who had good/excellent self-rated health (Table 1). Figure 1 shows the combined prevalence and 95% CI of good/excellent self-rated health for each diversity level estimated using multiple imputation.

**Table 1.** Characteristics of participants who had better self-rated health and who did not have better self-rated health

Characteristics^c^	Self-rated health, *n* (%)^a^		*P* value

	Poor/fair	Good/excellent	
Total	23,181	102,054	

Diversity level (range 0–4), mean (SD)^b^	0.78 (1.32)	1.37 (1.53)	<0.001

Each dimension of diversity			
Gender	4,347 (28)	32,728 (45)	<0.001
Residential area	1,830 (12)	16,745 (23)	<0.001
Age composition	2,083 (14)	17,076 (23)	<0.001

Age, mean (SD)	76 (6)	74 (6)	<0.001
Women	11,747 (51)	55,117 (54)	<0.001

Participating group			
Not involved in any groups	10,872 (71)	36,604 (52)	<0.001
Others	2,804 (18)	15,352 (22)	
Volunteer	293 (1.8)	2,441 (3.4)	
Hobby	1,295 (8.1)	8,925 (13)	
Sports	709 (4.4)	8,026 (11)	

Current workers	3,092 (15)	24,223 (26)	<0.001

Marital status			
Married	15,383 (68)	72,802 (73)	<0.001
Single	851 (3.8)	2,288 (2.3)	
Widowed/divorced	6,019 (27)	23,982 (24)	
Others	301 (1.3)	784 (0.8)	

Comorbidity of cancer	2,059 (9.0)	2,567 (2.7)	<0.001
Comorbidity of cardiac disease	4,705 (21)	8,547 (8.9)	<0.001
Comorbidity of stroke	1,556 (6.8)	2,562 (2.7)	<0.001

Equivalent annual household income			
<1.5 million yen	6,875 (38)	22,355 (27)	<0.001
1.5–2.4 million yen	6,850 (38)	34,252 (41)	
≥2.5 million yen	4,186 (23)	26,300 (32)	

Educational attainment			
<6 years	674 (3.0)	1,366 (1.4)	<0.001
6–9 years	10,743 (48)	38,586 (39)	
10–12 years	7,554 (34)	38,458 (38)	
≥13 years	3,516 (16)	21,673 (22)	

SD, standard deviation.

^a^The total numbers may vary across the characteristics because of missing data. The percentage may not total 100 because of rounding.

^b^We assessed three dimensions of the diversity (gender, residential area, and age composition) of the group in which each participant was involved most frequently. We then graded the diversity level into five categories: level 0 (not involved in any groups), level 1 (none of the three dimensions was diverse), level 2 (one of the three dimensions was diverse), level 3 (two of the three dimensions were diverse), level 4 (all dimensions were diverse).

^c^Data for 535 residential areas (school/community comprehensive care center districts) are not shown.

The PRs and 95% CIs for good/excellent self-rated health are shown in Table 2. The prevalence of good/excellent self-rated health was statistically significantly elevated by 1.03 times for every one unit increase in the diversity level (PR 1.03; 95% CI, 1.02–1.04). This is interpreted as meaning that the percentage increase in the prevalence of good/excellent self-rated health was approximately 13% in those with fully diverse group membership (level 4) compared with those not participating in any groups. A statistically significant increase in self-rated health was found in all diversity dimensions, with the association being more pronounced in the diversity in gender (PR 1.07; 95% CI, 1.04–1.09) (Table 2).

**Table 2.** Combined prevalence ratios and 95% confidence intervals for better self-rated health estimated by Poisson regression and robust variance with multiply imputed data

Characteristics	Prevalence ratio (95% CI)			

	Model 1^a^	*P* value	Model 2^b^	*P* value
Diversity level (range 0–4)	1.03 (1.02–1.04)	<0.001		

Each dimension of diversity				
Gender			1.07 (1.04–1.09)	0.002
Residential area			1.03 (1.02–1.04)	<0.001
Age composition			1.01 (1.00–1.02)	0.02

Age	0.99 (0.99–0.99)	<0.001	0.99 (0.99–0.99)	<0.001
Women	1.02 (1.02–1.03)	<0.001	1.02 (1.02–1.03)	<0.001
Current workers	1.07 (1.07–1.08)	<0.001	1.07 (1.07–1.08)	<0.001

Marital status				
Married	Reference		Reference	
Single	0.95 (0.92–0.97)	<0.001	0.95 (0.92–0.97)	<0.001
Widowed/divorced	1.01 (1.00–1.01)	0.10	1.01 (1.00–1.01)	0.12
Others	0.92 (0.89–0.95)	<0.001	0.92 (0.89–0.95)	<0.001

Cancer	0.70 (0.68–0.71)	<0.001	0.70 (0.68–0.71)	<0.001
Cardiac disease	0.81 (0.80–0.82)	<0.001	0.81 (0.80–0.82)	<0.001
Stroke	0.81 (0.78–0.83)	<0.001	0.81 (0.78–0.83)	<0.001

Income				
<1.5 million yen	Reference		Reference	
1.5–2.4 million yen	1.06 (1.05–1.07)	<0.001	1.06 (1.05–1.07)	<0.001
≥2.5 million yen	1.08 (1.07–1.08)	<0.001	1.08 (1.07–1.08)	<0.001

Education				
<6 years	Reference		Reference	
6–9 years	1.09 (1.05–1.12)	<0.001	1.08 (1.05–1.12)	<0.001
10–12 years	1.12 (1.08–1.15)	<0.001	1.12 (1.08–1.15)	<0.001
≥13 years	1.14 (1.10–1.17)	<0.001	1.14 (1.10–1.17)	<0.001

CI, confidence interval.

^a^Prevalence ratios of the diversity level estimated by Poisson regression with robust variance, adjusted for age, gender, current workers, marital status, comorbidities, household income, educational attainment, and residential area.

^b^Prevalence ratios of each dimension of diversity estimated by Poisson regression with robust variance, adjusted for age, gender, current workers, marital status, comorbidities, household income, educational attainment, and residential area.

When stratifying the analyses by type of group, the percentage increase of good/excellent self-rated health was 3–4% per one unit increase in the diversity level; the percentage increase for gender diversity was 5–12% (Table 3). Although the values are comparable, both PR scales of the diversity level and the diversity in gender were largest in sports groups (Table 3). The result of the sensitivity analysis with complete data was identical to the result with multiple imputation (eTable 2).

**Table 3.** Prevalence ratios and 95% confidence intervals for better self-rated health stratified by groups

Characteristics	Prevalence ratio (95% CI)			

	Model 1^a^	*P* value	Model 2^b^	*P* value
**Sports group**				
Diversity level (range 0–4)	1.04 (1.04–1.04)	<0.001		
Each dimension of diversity				
Gender			1.12 (1.10–1.13)	<0.001
Residential area			1.02 (1.01–1.04)	0.003
Age composition			1.01 (0.99–1.02)	0.21

**Hobby group**				
Diversity level (range 0–4)	1.03 (1.03–1.03)	<0.001		
Each dimension of diversity				
Gender			1.06 (1.05–1.07)	<0.001
Residential area			1.04 (1.03–1.06)	<0.001
Age composition			1.02 (1.01–1.04)	0.008

**Volunteer group**				
Diversity level (range 0–4)	1.03 (1.03–1.04)	<0.001		
Each dimension of diversity				
Gender			1.12 (1.09–1.15)	<0.001
Residential area			1.01 (0.97–1.05)	0.57
Age composition			0.97 (0.94–1.00)	0.10

**Other group**				
Diversity level (range 0–4)	1.03 (1.03–1.03)	<0.001		
Each dimension of diversity				
Gender			1.09 (1.08–1.10)	<0.001
Residential area			1.01 (0.99–1.02)	0.25
Age composition			1.00 (0.99–1.02)	0.73

CI, confidence interval.

^a^Prevalence ratios of the diversity level stratified by groups, estimated by Poisson regression with robust variance, adjusted for age, gender, current workers, marital status, comorbidities, household income, educational attainment, and residential area.

^b^Prevalence ratios of each dimension of the diversity stratified by groups, estimated by Poisson regression with robust variance, adjusted for age, gender, current workers, marital status, comorbidities, household income, educational attainment, and residential area.

## DISCUSSION

Among the older population in Japan, we found that higher levels of group diversity were associated with members’ better self-rated health; the increase in the prevalence of good/excellent self-rated health by one-unit increment of the diversity level was estimated to be 3%. We also found that the key dimension associated with better self-rated health was the diversity in gender. These associations were consistent across the group types but potentially most pronounced in sports groups.

The reason better self-rated health was associated with the diversity of group membership may be explained by the concept of social capital, and in particular, bridging social capital.^7^^,^^18^ Participants involved in a group with diverse membership (ie, heterogeneous membership) might have more opportunities to expand their social capital. Increased bridging social capital may provide them multiple resources for better health.^1^^–^^3^ Given that the key dimension associated with health was gender diversity, resources potentially provided by the members of the opposite gender may be more valuable compared with those brought by the members from other areas or different age groups. A study by Westermann et al showed that the frequency of requiring help to solve a problem in groups with mixed gender (21.9%) was lower compared with that of men’s only groups (33.3%) or women’s only groups (25.0%), suggesting that gender diversity is a key factor determining the maturity and solidarity of the group.^19^

Previous studies from the JAGES have suggested that participating in sports groups is protective for health.^2^^–^^4^ Other cohort studies have also suggested that regular physical exercise has protective effects against dementia, diabetes, and mortality.^20^^–^^22^ In the present study, the association was largest in sports groups. Kanamori et al demonstrated that the risk of incident functional disability was lower among seniors only participating in sports organizations but not doing exercise regularly compared with those not participating in any sports organizations but doing exercise alone,^3^ suggesting that a sports group is an efficient place to form social connections to protect health. According to these findings, we were able to assume that the protective nature of sports groups may empower their participants via the diversity of gender in their membership.

A strength of our study is the large sample size. Because of this advantage regarding statistical power, we were able to create more categories of diversity levels compared to recent smaller studies^8^ and provide gender-stratified analysis, holding a sufficient sample size within each stratum. Also, this is the first study to demonstrate that the diversity in gender was the essential (but not exclusive) dimension for better health in group participation. Our study population in Japan may also contribute to the new findings. Japanese society is relatively closed or homogeneous compared with Western societies^8^; therefore, the health status of the older population would be more sensitive to diversity. In addition, older people in Japan may be sensitive to gender diversity because of the traditionally strict gender roles.

This study has implications for public health. The potential impact of group diversity (13% more good self-rated health) is about the same strength of association as other important social determinants, such as education.^7^ Community interventions to facilitate social groups or social gathering opportunities^23^ should attempt to invite participants with various backgrounds, especially in terms of gender. However, because the potential impact of group diversity is not very large, those interventions should find cost-effective ways in practice.

Our study has limitations. First, this cross-sectional study could not definitively conclude a causative effect of diversity of group membership on improvement in self-rated health. There might be reverse causation, in which healthier participants would have more opportunities to participate in groups with more diverse membership. Also, there could be a measurement bias; for example, a higher diversity level in the dimension of the area of residence might indicate a higher mobility function of respondents as well as other members. Further studies are needed to address this limitation. Second, even though we tried to attempt multiple imputation for missing data using a chain equation including all dependent and independent variables, the fact that one-third of the information on diversity was missing might be a concern. However, background characteristics did not differ between those with complete data and those with missing data. The result of a sensitivity analysis was identical to the final result with multiple imputation. Thus, this limitation may not affect the conclusion. Finally, because of the limited available data, we could only assess diversity in the groups in which the participants participated most frequently, and we could not evaluate the effect of diversity in other groups. Participation in multiple groups was associated with better health, and this effect has been shown to be dose-dependent in previous studies.^2^^,^^8^ It is plausible that older adults who participate in multiple groups may receive more benefit through their multiple participation compared with those who participate in only one group. In addition, we did not include different weights of the each diversity dimension for the composite measure (the continuous variable) of the overall diversity levels, and some trends were somewhat nonlinear in the present study. Thus, we need further studies focusing on the association between health and the diversity in multiple group participations, as well as other dimensions of diversity (eg, social class)^24^ and other measurements of the diversity (eg, a categorical variable) we could not evaluate in the present study.

In summary, higher diversity of group membership is associated with better self-rated health, and gender diversity is the key dimension associated with better health. Further studies are needed to determine the association between diversity in a group and better health in older populations.

## APPENDIX A. SUPPLEMENTARY DATA

eTable 1. Characteristics of those who completed the information on diversity and those who did not

eTable 2. Sensitivity analysis of participants with complete data
