# Supplementary material for: Participation in Community Group Activities Among Older Adults: Is Diversity of Group Membership Associated With Better Self-rated Health?
Source: J Epidemiol. 2018 Nov 5;28(11):452–7. doi: 10.2188/jea.JE20170152 (PMC6192976; doi:10.2188/jea.JE20170152)
Supplement: Supplementary file 1 [file je-28-452-s001.pdf]

eTable 1. Characteristics of those who completed the information on diversity and those who did not

Characteristics	Incomplete	Complete
Total, n (%)	48,084 (100)	81,656 (100)
Age, mean (SD)	75 (6)	74 (6)
Women, n (%)	28,168 (59)	41,450 (51)
Self-rated health, n (%)		
Poor/fair	8,610 (18)	14,571 (18)
Good/excellent	37,173 (77)	64,881 (79)
Missing	2,301 (4.8)	2,204 (2.7)
Current workers, n (%)	9,811 (20)	18,243 (22)
Missing	7,807 (16)	5,490 (6.7)
Marital status, n (%)		
Married	31,774 (66)	58,980 (72)
Single	2,318 (4.8)	1,192 (1.5)
Widowed/divorced	12,646 (26)	18,770 (23)
Others	433 (0.9)	710 (0.9)
Missing	913 (1.9)	2,004 (2.5)
Comorbidity of cancer, n (%)	1,623 (3.4)	3,116 (3.8)

Comorbidity of cardiac disease, n (%)	5,069 (11)	8,579 (11)
Comorbidity of stroke, n (%)	1,594 (3.3)	2,642 (3.2)
Missing comorbidity, n (%)	4,119 (8.6)	4,548 (5.6)
Equivalent annual household income, n (%)		
<1.5 million yen	12,202 (25)	18,119 (22)
1.5–2.4 million yen	13,776 (29)	28,383 (35)
≥2.5 million yen	9,197 (19)	21,970 (27)
Missing	12,909 (27)	13,184 (17)
Educational attainment, n (%)		
<6 years	974 (2.0)	1,195 (1.5)
6–9 years	21,406 (45)	30,021 (37)
10–12 years	16,091 (33)	31,199 (38)
≥13 years	7,947 (17)	17,920 (22)
Missing	1,666 (3.5)	1,321 (1.6)

SD, standard deviation.

[a] The percentage may not total 100 because of rounding.

eTable 2. Sensitivity analysis of participants with complete data

	Prevalence ratio (95% CI)			
**Characteristics**	**Model 1**	**P value**	**Model 2**	**P value**
**Overall**				
Diversity level (range, 0–4)	1.03 (1.03–1.04)	<0.001		
Diverse dimension				
Gender			1.08 (1.08–1.09)	<0.001
Residential area			1.03 (1.02–1.04)	<0.001
Age composition			1.00 (0.99–1.01)	0.64
**Stratified by groups**				
**Sports group**				
Diversity level (range, 0–4)	1.04 (1.04–1.04)	<0.001		
Diverse dimension				
Gender			1.12 (1.10–1.13)	<0.001
Residential area			1.02 (1.00–1.04)	0.01
Age composition			1.01 (0.99–1.02)	0.44
**Hobby group**				
Diversity level (range, 0–4)	1.03 (1.03–1.04)	<0.001		
Diverse dimension				
Gender			1.05 (1.04–1.07)	<0.001
Residential area			1.05 (1.04–1.07)	<0.001

Age composition			1.02 (1.00–1.03)	0.06
**Volunteer group**				
Diversity level (range, 0–4)	1.03 (1.03–1.04)	<0.001		
Diverse dimension				
Gender			1.12 (1.09–1.15)	<0.001
Residential area			1.02 (0.99–1.05)	0.28
Age composition			0.96 (0.93–1.00)	0.04
**Other group**				
Diversity level (range, 0–4)	1.03 (1.03–1.03)	<0.001		
Diverse dimension				
Gender			1.09 (1.08–1.11)	<0.001
Residential area			1.01 (0.99–1.03)	0.29
Age composition			0.99 (0.98–1.01)	0.34

CI, confidence interval.

[a] Prevalence ratios of the diversity level estimated by Poisson regression with robust variance, adjusted for age, gender, current workers, marital status, comorbidities, household income, educational attainment, and residential area.

[b] Prevalence ratios of each dimension of the diversity estimated by Poisson regression with robust variance, adjusted for age, gender, current workers, marital status, comorbidities, household income, educational attainment, and residential area.
